# Chronotype as an Independent Predictor of Cardiovascular Risk in Euthymic Bipolar Patients

**DOI:** 10.1192/j.eurpsy.2026.11185

**Published:** 2026-07-31

**Authors:** I. Uysal, I. Keleş Altun

**Affiliations:** Department of Psychiatry, Bursa Yüksek İhtisas Training and Research Hospital, Bursa, Türkiye

## Abstract

**Introduction:**

Patients with bipolar disorder (BD) have an increased risk of cardiovascular morbidity and mortality compared to the general population. While metabolic and pharmacological factors are well-documented contributors, the role of circadian rhythm and chronotype in cardiovascular vulnerability remains underexplored.

**Objectives:**

To examine whether chronotype differences influence cardiovascular risk in euthymic bipolar patients and whether this effect remains independent of medication dose, mood stabilizer levels, illness duration, and other clinical variables.

**Methods:**

Sixty euthymic BD patients (30 morning-type, 30 evening-type) were evaluated. Euthymia was confirmed using the Young Mania Rating Scale (YMRS) and the Hamilton Depression Rating Scale (HDRS). Chronotype was assessed with the Morningness–Eveningness Questionnaire (MEQ). Cardiovascular risk was calculated using the QRISK3 algorithm, which integrates variables such as age, sex, blood pressure, smoking, diabetes, lipid profile, and BMI. The algorithm provides 10-year cardiovascular risk (%QRISK3), relative risk (RR), and heart age. The difference between heart and biological age was defined as the Heart–Biological Age Gap. Two multiple linear regression models were performed: a hierarchical regression for Relative Risk (RR), including MEQ score, illness duration, chlorpromazine-equivalent dose, and age at onset; and another model for the Heart–Biological Age Gap, including MEQ score, illness duration, chlorpromazine-equivalent dose, age at onset, and serum lithium and valproate levels.

**Results:**

Both QRISK3 and Relative Risk (RR) scores were elevated compared to population norms, indicating increased cardiovascular vulnerability among euthymic bipolar patients. Evening-type individuals had significantly higher RR (Z = –2.43, p = 0.015) and greater Heart–Biological Age Gap (Z = –2.39, p = 0.017) than morning-types.

In the regression model predicting RR, lower MEQ scores (reflecting greater eveningness) significantly predicted higher RR (β = –0.009, p = 0.003; F = 5.00, p = 0.003), while other clinical and pharmacological variables were not significant.

In the model predicting the Heart–Biological Age Gap, both lower MEQ scores (β = –0.156, p = 0.001; F = 5.63, p < 0.001) and higher chlorpromazine-equivalent doses (β = 0.012, p = 0.003) were associated with greater cardiovascular aging.

**Image 1:**
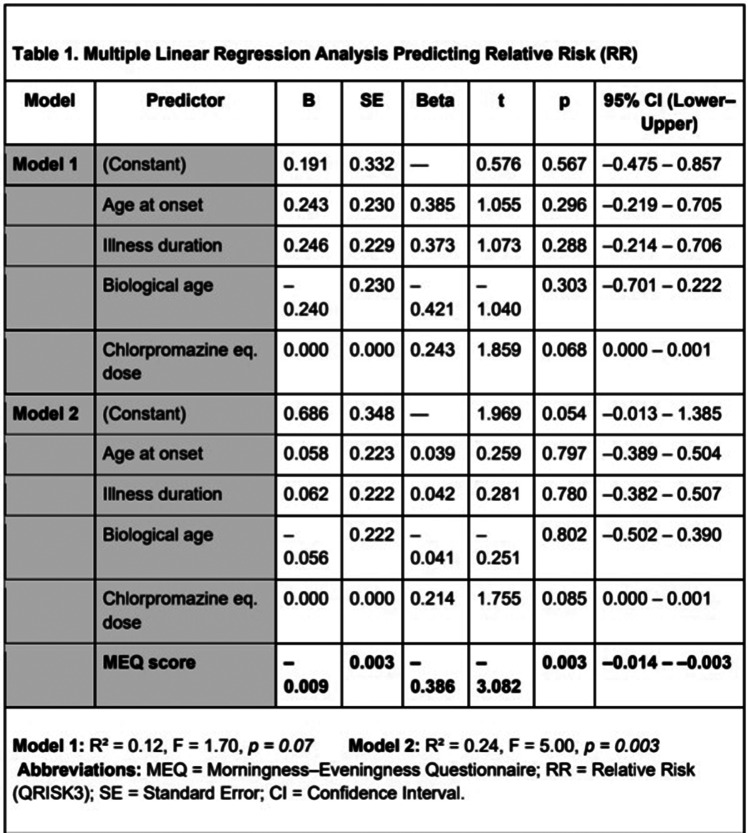
Image 1: Long description.

**Image 2:**
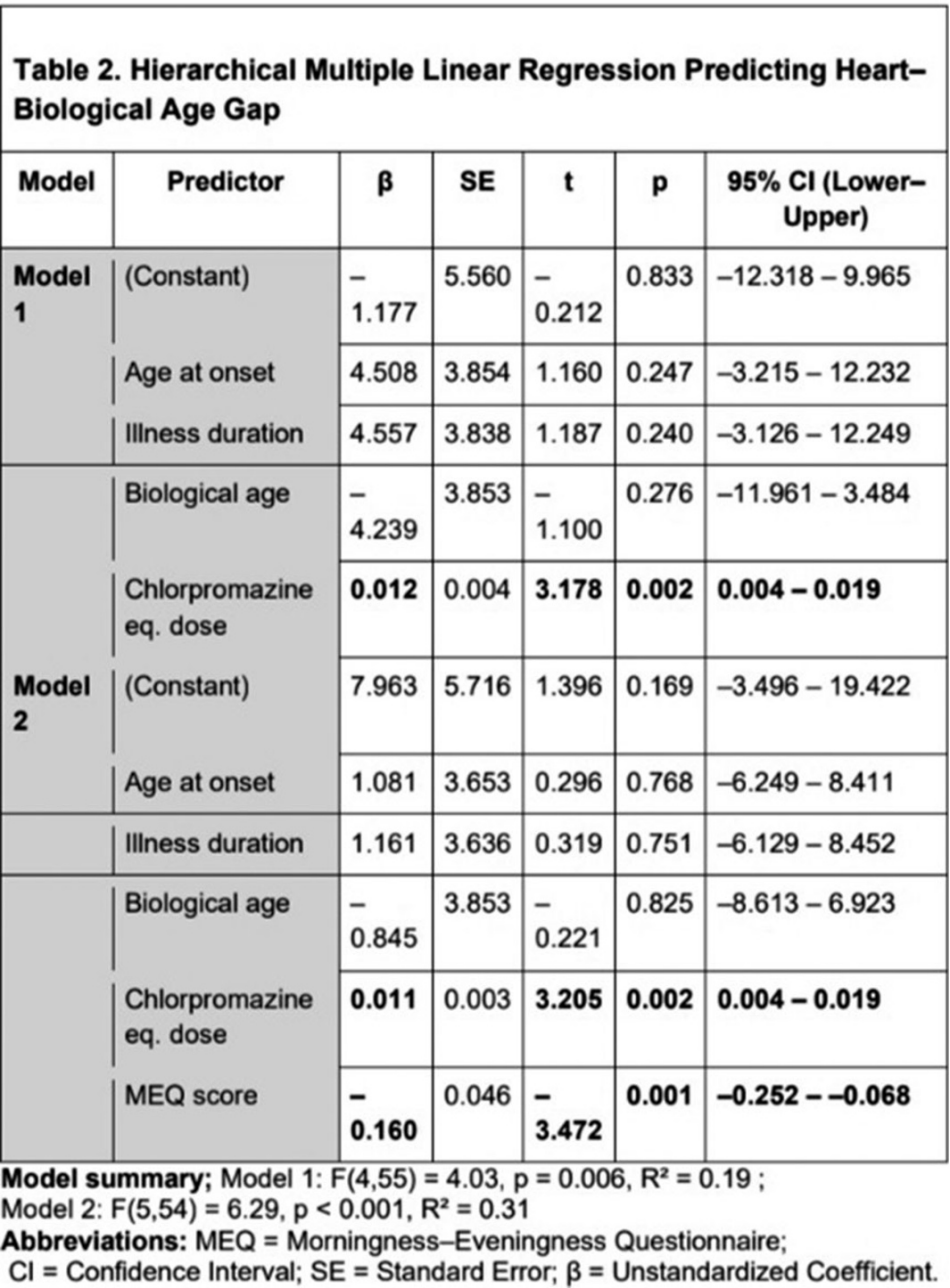
Image 2: Long description.

**Conclusions:**

Chronotype appears to represent a clinically meaningful biological marker of cardiovascular vulnerability in bipolar disorder. Evening-type patients displayed greater cardiovascular risk and accelerated vascular aging even during euthymic periods, independent of medication dose or illness variables. Clinicians should consider chronotype assessment as an early warning signal for latent cardiometabolic risk. Integrating circadian evaluation into psychiatric follow-up may enhance prevention and long-term outcomes in bipolar disorder.

**Disclosure of Interest:**

None Declared

## Long descriptions

### Image 1: Long description

The table is titled Table 1. Multiple Linear Regression Analysis Predicting Relative Risk R R. It contains columns for Model, Predictor, B, S E, Beta, t, p, and 95% C I (Lower–Upper).

Model 1 results:

* (Constant): B 0.191, S E 0.332, t 0.576, p 0.567, C I -0.475 to 0.857.

* Age at onset: B 0.243, S E 0.230, Beta 0.385, t 1.055, p 0.296, C I -0.219 to 0.705.

* Illness duration: B 0.246, S E 0.229, Beta 0.373, t 1.073, p 0.288, C I -0.214 to 0.706.

* Biological age: B -0.240, S E 0.230, Beta -0.421, t -1.040, p 0.303, C I -0.701 to 0.222.

* Chlorpromazine eq. dose: B 0.000, S E 0.000, Beta 0.243, t 1.859, p 0.068, C I 0.000 to 0.001.

Model 2 results:

* (Constant): B 0.686, S E 0.348, t 1.969, p 0.054, C I -0.013 to 1.385.

* Age at onset: B 0.058, S E 0.223, Beta 0.039, t 0.259, p 0.797, C I -0.389 to 0.504.

* Illness duration: B 0.062, S E 0.222, Beta 0.042, t 0.281, p 0.780, C I -0.382 to 0.507.

* Biological age: B -0.056, S E 0.222, Beta -0.041, t -0.251, p 0.802, C I -0.502 to 0.390.

* Chlorpromazine eq. dose: B 0.000, S E 0.000, Beta 0.214, t 1.755, p 0.085, C I 0.000 to 0.001.

* M E Q score (bolded): B -0.009, S E 0.003, Beta -0.386, t -3.082, p 0.003, C I -0.014 to -0.003.

Footer data:

Model 1: R super 2 = 0.12, F = 1.70, p = 0.07.

Model 2: R super 2 = 0.24, F = 5.00, p = 0.003.

Abbreviations: M E Q = Morningness–Eveningness Questionnaire; R R = Relative Risk (QRISK3); S E = Standard Error; C I = Confidence Interval.

Navigate back to Image 1:.

### Image 2: Long description

The table contains seven columns: Model, Predictor, beta (unstandardized coefficient), S E (standard error), t, p, and 95% C I (Lower-Upper).

Model 1 results:

* (Constant): beta -1.177, S E 5.560, t -0.212, p 0.833, C I -12.318 to 9.965.

* Age at onset: beta 4.508, S E 3.854, t 1.160, p 0.247, C I -3.215 to 12.232.

* Illness duration: beta 4.557, S E 3.838, t 1.187, p 0.240, C I -3.126 to 12.249.

* Biological age: beta -4.239, S E 3.853, t -1.100, p 0.276, C I -11.961 to 3.484.

* Chlorpromazine eq. dose: beta 0.012, S E 0.004, t 3.178, p 0.002, C I 0.004 to 0.019.

Model 2 results:

* (Constant): beta 7.963, S E 5.716, t 1.396, p 0.169, C I -3.496 to 19.422.

* Age at onset: beta 1.081, S E 3.653, t 0.296, p 0.768, C I -6.249 to 8.411.

* Illness duration: beta 1.161, S E 3.636, t 0.319, p 0.751, C I -6.129 to 8.452.

* Biological age: beta -0.845, S E 3.853, t -0.221, p 0.825, C I -8.613 to 6.923.

* Chlorpromazine eq. dose: beta 0.011, S E 0.003, t 3.205, p 0.002, C I 0.004 to 0.019.

* M E Q score: beta -0.160, S E 0.046, t -3.472, p 0.001, C I -0.252 to -0.068.

Model Summary:

Model 1: F(4,55) = 4.03, p = 0.006, R-squared = 0.19.

Model 2: F(5,54) = 6.29, p < 0.001, R-squared = 0.31.

Abbreviations: M E Q = Morningness-Eveningness Questionnaire; C I = Confidence Interval; S E = Standard Error; beta = Unstandardized Coefficient.

Navigate back to Image 2:.

